# Advancing Understanding of Epilepsy and Type 1 Diabetes Mellitus: A Global Perspective on Research Trends and Future Directions

**DOI:** 10.1155/jdr/8836992

**Published:** 2025-10-07

**Authors:** Ruirui Zhang, Shenglin Wang, Sirui Chen, Junqiang Li, Dadong Luo, Kaiyun Jia, Yihe Lian, Tiancheng Wang, Xin Tian

**Affiliations:** ^1^Department of Neurology, Epilepsy Center, The Second Hospital & Clinical Medical School, Lanzhou University, Lanzhou, China; ^2^Department of Neurology, The First Affiliated Hospital of Chongqing Medical University, Chongqing Key Laboratory of Major Neurological and Mental Disorders, Chongqing, China; ^3^Department of Epilepsy Center, The First Affiliated Hospital of Chongqing Medical University, Chongqing, China; ^4^Department of Geriatrics, Laboratory of Research and Translation for Geriatric Diseases, The First Affiliated Hospital of Chongqing Medical University, Chongqing, China; ^5^Key Laboratory of Major Brain Disease and Aging Research (Ministry of Education), Chongqing Medical University, Chongqing, China

**Keywords:** bibliometrics, databases, genetic, diabetes mellitus, Type 1, epilepsy, gene expression profiling, genetic association studies, TSPO protein, human

## Abstract

**Aims:**

Evidence suggests a bidirectional relationship between epilepsy and Type 1 diabetes mellitus (T1DM), but the underlying mechanisms and overall research landscape remain incompletely understood. This study is aimed at delineating research trends and sharing pathogenic pathways between epilepsy and T1DM through a comprehensive bibliometric analysis and genetic investigation.

**Methods:**

We performed a systematic search of the Web of Science Core Collection database (from 1983 to 2024) to identify relevant publications on epilepsy and T1DM. Bibliometric tools (Bibliometrix and CiteSpace) were employed to analyze publication trends, major contributors, and research hotspots. Shared genes were identified via disease–gene association databases (GeneCards and OMIM), and differentially expressed genes (DEGs) were analyzed using GEO2R on GEO datasets.

**Results:**

A total of 217 publications were included. Publication output demonstrated exponential growth over the study period (*R*^2^ = 0.6999). The United States, the United Kingdom, and China were the leading contributing countries, exhibiting varying collaboration patterns. Analysis of keywords and co-citations highlighted the central role of autoimmunity, particularly glutamic acid decarboxylase (GAD) antibodies, in linking the two diseases. Keyword and thematic analyses revealed that recent trends indicate growing attention to clinical management, particularly of severe hypoglycemia. Genetic analysis identified 44 overlapping genes between epilepsy and T1DM, which were significantly enriched for autoimmune-associated terms (*p* < 0.001). Notably, the translocator protein (TSPO) was the only DEG in both conditions, with upregulation observed in T1DM (log2FC = 0.504, *p* = 0.0319) and epilepsy (log2FC = 0.562, *p* < 0.001).

**Conclusions:**

This study maps the evolving research landscape of epilepsy and T1DM, confirming autoimmunity as a key link. The identification of TSPO as a shared, upregulated gene provides novel molecular evidence for the connection between the two diseases and suggests TSPO as a potential target for future research and therapeutic strategies.

## 1. Introduction

Epilepsy and Type 1 diabetes mellitus (TIDM) are distinct chronic conditions that significantly impact global health. Epilepsy affects over 70 million individuals worldwide, characterized by recurrent, unprovoked seizures stemming from abnormal neuronal activity [1]. T1DM, an autoimmune disorder that leads to pancreatic *β*-cell destruction and insulin deficiency, affects an estimated 8.4 million individuals globally [2]. Although epilepsy and T1DM have traditionally been considered distinct entities with differing pathophysiological mechanisms, recent epidemiological evidence increasingly supports a bidirectional association.

Population-based studies have reported a two- to three-fold increased risk of epilepsy in individuals with T1DM compared to the general population [3–5]. Additionally, a recent Mendelian randomization study suggested that patients with T1DM may have a heightened risk of developing epilepsy [6]. Conversely, research has identified a four-fold higher prevalence of T1DM among patients with idiopathic generalized epilepsy [7]. These epidemiological findings suggest that shared pathogenic mechanisms—such as autoimmunity, metabolic dysregulation, genetic susceptibility, and cerebrovascular injury—may underlie the comorbidity [8, 9]. Notably, the comorbidity of epilepsy and T1DM not only complicates clinical management but also potentially leads to adverse health outcomes. Seizures and the use of antiseizure medications may exacerbate metabolic disturbances in patients with T1DM, while the significant blood glucose fluctuations or hypoglycemic events can further trigger seizures, thereby affecting patients' quality of life and prognosis [10–12]. Thus, a comprehensive understanding of the relationship between epilepsy and T1DM, including the underlying comorbidity mechanisms, is crucial for developing effective intervention strategies.

Nevertheless, research on this comorbidity faces limitations. First, mechanistic insights remain fragmented. Although hypotheses have implicated immune dysregulation, metabolic dysfunction, and genetic predisposition, genetic evidence remains scarce [8, 9]. Existing data predominantly derive from single-disease mutation screens and lack systematic cross-disease validation. Second, most studies rely on clinical observations (e.g., retrospective cohort analyses and case reports). While some reviews and meta-analyses exist, no bibliometric study has yet objectively quantified the field's evolution, collaborative networks, and emerging hotspots.

Bibliometrics—an emerging interdisciplinary discipline grounded in statistics and mathematics—provides powerful tools for the quantitative analysis of literature, mapping of research landscapes, identification of key contributors, and detection of emerging trends [13]. To fill this gap, we present the first comprehensive bibliometric analysis (1983–2024) of epilepsy–T1DM comorbidity research using CiteSpace to uncover latent trends and hotspots. We further identify shared genetic risk factors by integrating data from disease–gene association databases (GeneCards and OMIM) and GEO datasets. Collectively, this work offers a data-driven foundation for future mechanistic studies and clinical interventions.

## 2. Methods

### 2.1. Data Source and Search Strategy

The Web of Science is an authoritative database that helps researchers identify research hotspots, trends, and knowledge structures in their fields. For this study, publication data related to T1DM and epilepsy were extracted from the Web of Science Core Collection (WoSCC) to conduct an in-depth bibliometric analysis. To ensure the accuracy and timeliness of the data, all search, data extraction, and download processes were completed on the same day, March 8, 2025. Detailed screening procedures and the complete search strategy are provided in Figure S1 and Table S1.

### 2.2. Bibliometric Analysis

The bibliometric analysis was conducted using the R Bibliometrix package (Version 4.3.0) [14] and CiteSpace (Version 6.4) [15]. These tools were used to examine publication trends, major contributors (countries, institutions, authors, and journals), keyword trends, citation clustering, and burst cycles in the field of epilepsy and T1DM. They were employed for visualization analysis to gain insights into the field and uncover research frontiers through large-scale data.

### 2.3. Genetic Overlap Analysis

To investigate genetic connections between epilepsy and T1DM, we employed a multifaceted approach: (1) *disease–gene association identification*: We used GeneCards (https://www.genecards.org/) and OMIM databases (https://www.omim.org/) to identify genes associated with epilepsy and T1DM, setting a relevance score threshold of 10 for GeneCards entries; (2) *overlapping gene analysis*: We identified and visualized the intersection of genes associated with both conditions using TBtools [16]; (3) *hub gene identification*: Using Cytoscape (Version 3.10.1)[17] with the Maximal Clique Centrality (MCC) algorithm, we ranked the Top 10 hub genes among the overlapping genes based on the protein-protein interaction (PPI) network constructed by the STRING online database (https://string-db.org/); (4) *functional enrichment analysis*: Disease Ontology (DO), Gene Ontology (GO) and Kyoto Encyclopedia of Genes and Genomes (KEGG) pathway analyses were conducted using the clusterProfiler package in R [18], with *p* values adjusted by the Benjamini–Hochberg correction (statistically significant at *p* < 0.05); (5) *differential gene expression analysis*: GEO datasets (https://www.ncbi.nlm.nih.gov/geo/, GSE123658 for T1DM and GSE143272 for epilepsy) were analyzed using GEO2R to identify DEGs with significance thresholds of *p* < 0.05 and |log2FC| > 0.5. The overlap between DEGs in epilepsy and T1DM was determined and visualized.

## 3. Results

### 3.1. Publication Trends and Growth Analysis

**Figure 1:**
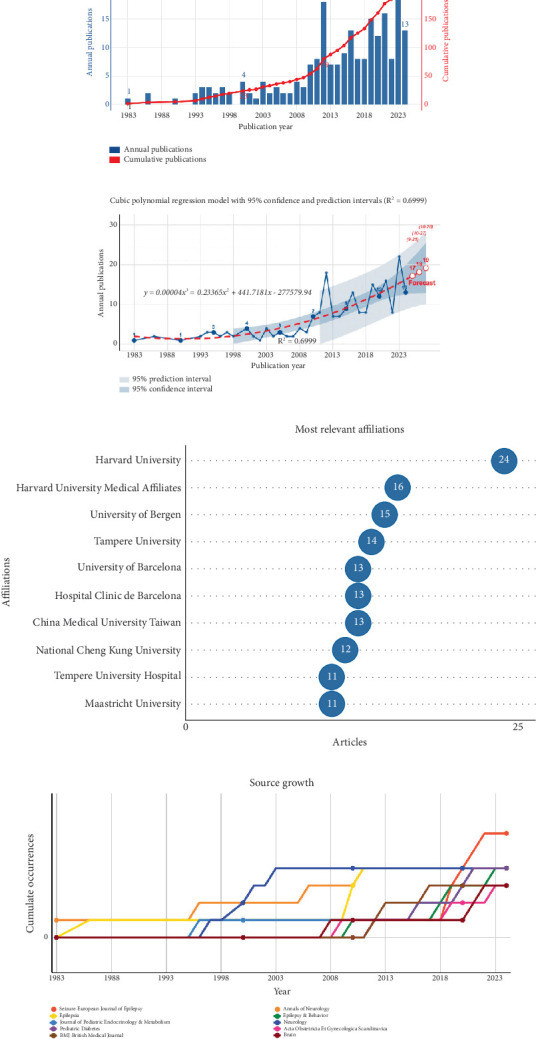
(a–d) Publication trends, leading affiliations, and primary sources in the epilepsy and T1DM research field from 1983 to 2024.

Publications on the topic of epilepsy and T1DM have significantly increased over the past four decades (Figure 1). The first documented publication appeared in 1983, with minimal research activity until the mid-1990s. After 2009, there has been a substantial acceleration in publication output. The past decade (2015–2024) accounted for 57.1% of all publications, with 2023 marking the highest annual output (22 publications), which is nearly 10 times more than in 1993. Trend analysis using cubic polynomial regression (*R*^2^ = 0.6999) indicated a continued growth trajectory in this research field (Figure 1). This suggests that, over time, an increasing number of scholars will focus on this field.

### 3.2. Distribution of Publications by Country and Institution

Analysis of country contributions revealed the United States as the leading contributor with 37 publications (17.1%), followed by the United Kingdom (23 publications, 10.6%) and China (15 publications, 6.9%) (Table 1). European countries demonstrated a strong presence, with Italy (6.5%), France (5.5%), Germany (4.6%), and the Netherlands (3.2%) among the Top 10 contributors. International collaboration patterns varied considerably among countries. While the Netherlands ranks ninth in total publications, it has the highest proportion of international collaborations (57.1%). In contrast, Norway showed no international collaborations, with all publications originating from single-country research teams. This lack of international cooperation has limited progress in epilepsy–T1DM research and warrants attention.

Among the Top 10 most relevant affiliations in terms of publication output, each institution published over 10 articles, highlighting their active engagement in this field. Notably, Harvard University led with 24 publications, demonstrating its outstanding research capabilities (Figure 1c).

### 3.3. Author Contributions and Citation Impact

Between 1983 and 2024, the field of epilepsy and T1DM produced a number of highly productive and influential researchers (Table 2). Graus F. had five publications with 935 citations, while Saiz A. had similar productivity (five publications) but with an even higher citation impact (1045 citations). Both researchers achieved remarkable results and gained widespread recognition. Although other Top 10 authors had fewer publications, each published at least two papers. Collectively, they have driven academic progress in this field.

### 3.4. Analysis of Journal Distribution

Since 1983, there has been a steady increase in the number of publications on epilepsy and T1DM, with 156 journals contributing articles (Figure 1). *Annals of Neurology* published the first article in this field. *Neurology* has seen a intermittent increase in publications from 1996 to 2003. *Seizure-European Journal of Epilepsy* started later but saw a substantial increase in publications after 2018, surpassing other journals by 2021 to become the journal publishing the most articles in this field. Additionally, high-impact journals such as the *British Medical Journal* and *Brain* have each published three papers in this field.

### 3.5. Most Influential Publications

Analysis of highly cited papers identified seminal publications that have shaped the understanding of the epilepsy–T1DM relationship (Table 3). The most influential publication was “Autoantibodies to Glutamic Acid Decarboxylase in Patients With Therapy-Resistant Epilepsy” by Peltola et al. (*Neurology*, 2000), with 27 local citations [19]. This pioneering study established the association between glutamic acid decarboxylase (GAD) autoimmunity and epilepsy, particularly therapy-resistant temporal lobe epilepsy, providing a foundational link to the pathophysiology of epilepsy–T1DM. Notably, several studies (ranked 2nd, 3rd, 5th, 6th, 7th, and 9th) have focused on the epidemiological association between epilepsy and T1DM, suggesting that they may share a common pathophysiological mechanism. Additionally, studies on the role of GAD antibodies in various neurological syndromes (ranked 4th and 8th) and the autoimmune mechanisms underlying epilepsy (ranked 10th) also laid the foundation for the field.

### 3.6. Changing Trends in Research Disciplines

**Figure 2:**
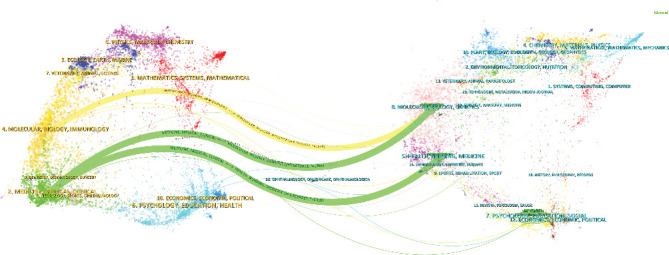
The dual-map overlay and corresponding disciplines.

To thoroughly investigate the interdisciplinary citation relationships between epilepsy and T1DM, this study employed a dual-map overlay analysis.  Figure 2 displays two distinct citation networks: the left side shows citing journals, while the right side shows cited journals. The thickest lines indicate three core citation paths. The yellow path signifies that articles on epilepsy and T1DM published in molecular/biology/immunology journals frequently cite those in molecular/biology/genetics journals. The green path indicates that articles published in medicine/medical/clinical journals commonly cite those in health/nursing/medicine and molecular/biology/genetics journals.

### 3.7. Co-occurrence Analysis

**Figure 3:**
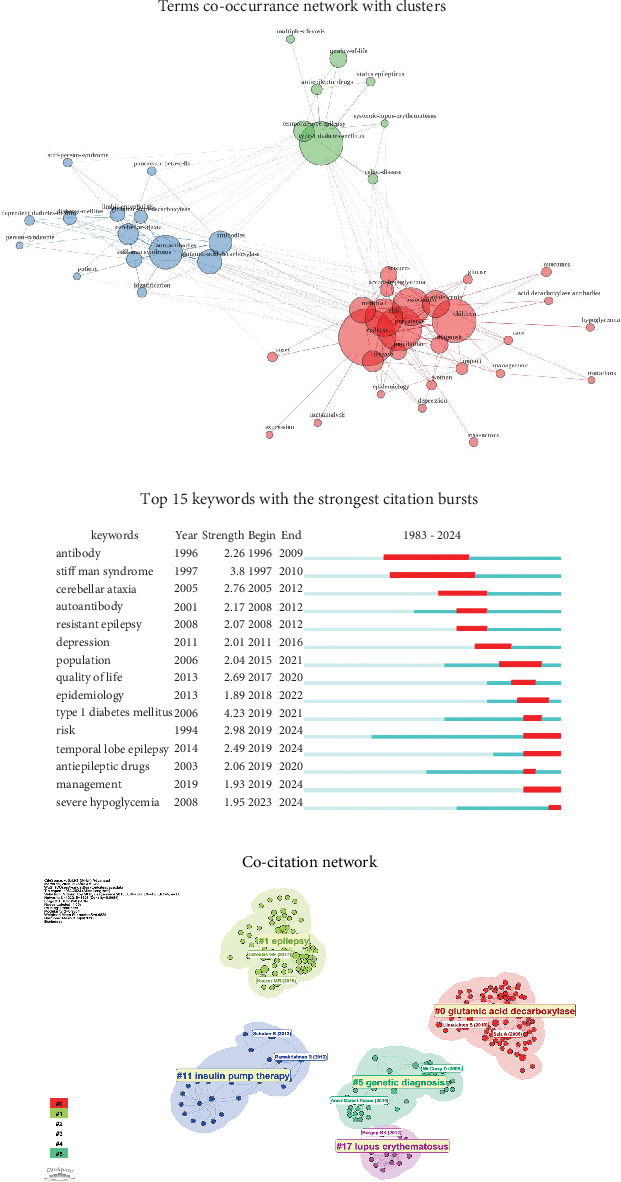
Visualized analysis of keywords and literature related to epilepsy and T1DM. (a) The terms co-occurrence network of the 217 documents. Nodes represent keywords. Lines refer to the co-occurring relationship. (b) Burst strength and time duration of the Top 15 keywords with the strongest citation bursts. (c) The major clusters and references of the co-citation network.

Keyword co-occurrence analysis is an effective method in bibliometrics for revealing hot topics and knowledge structures within a research field. We conducted a co-occurrence analysis of keywords in the field of epilepsy and T1DM (Figure 3). The graph is centered on T1DM and epilepsy. The red cluster primarily involves epidemiological studies on “risk”, “prevalence”, “children”, and “adolescents”. The blue cluster focuses on immune-related pathological mechanisms and comorbidities such as “autoantibodies”, “glutamic acid decarboxylase”, “cerebellar ataxia”, “stiff man syndrome”, and “limbic encephalitis”. The green cluster centers on “temporal lobe epilepsy”, “antiepileptic drugs”, and “quality of life” as well as other clinical interventions and outcomes. Epilepsy and T1DM exhibit clear shared pathways indicating commonalities in epidemiology, pathogenesis, clinical comorbidities, and interventions.

We also conducted burst analysis for keywords (Figure 3b). Early studies focused on autoimmune neurological syndromes (“stiff man syndrome” and “cerebellar ataxia”), marking the discovery of autoantibodies (e.g., GAD antibodies) in neurological diseases. The focus then shifted to epidemiology and clinical manifestations (“resistant epilepsy”, “depression”, and “quality of life”). Recently, the emphasis has been on clinical risk assessment and management (“risk”, “temporal lobe epilepsy”, and “management”), with particular attention to the impact of metabolic disturbances, such as severe hypoglycemia, on epilepsy.

### 3.8. Co-citation Analysis

Two papers were jointly cited by another publication, indicating a co-citation relationship. Based on this approach, CiteSpace was employed to construct a generalized co-citation network, which enables the identification of key publications, knowledge bases, and research frontiers within the field. As shown in Figure 3, the co-cited literature is synthesized into five large clusters, with the representative literature in each cluster displayed as “author's name (year)”. The representative publications in Cluster 1 established the epidemiological nexus between epilepsy and T1DM through retrospective cohort studies, elucidating a bidirectional, mutually reinforcing relationship. In Cluster 0, the representative publications delineated the role of GAD antibodies in temporal lobe epilepsy and an array of neurologic syndromes, highlighting the bridging function of GAD antibodies. In Cluster 5, McCorry D. first reported the increased prevalence of T1DM in patients with idiopathic generalized epilepsy, suggesting the possibility of shared susceptibility genes. In Cluster 11, insulin pump therapy reduced the occurrence of severe hypoglycemic events and the risk of seizures by providing more precise insulin delivery. In Cluster 17, studies have shown that the risk of epilepsy is elevated in systemic autoimmune diseases, with the highest risk observed in systemic lupus erythematosus.

### 3.9. Thematic Structure and Evolution of the Research Field

**Figure 4:**
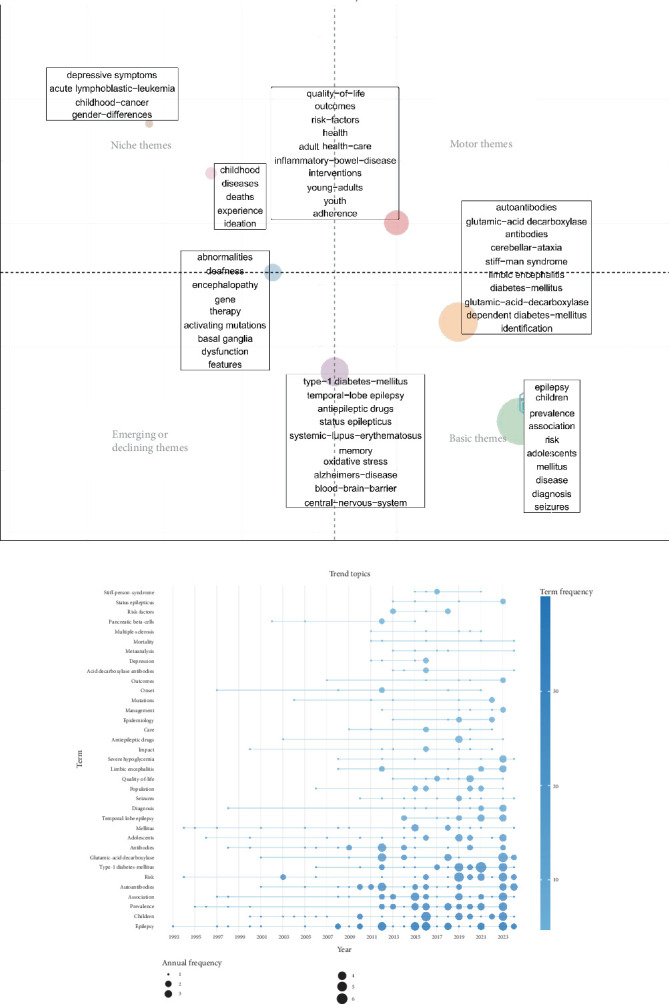
Analysis of thematic words and trend topics. (a) Thematic analysis in the field of epilepsy and T1DM. The horizontal and vertical axes represent centrality and density, respectively. (b) Timeline of research trends in the field of epilepsy and T1DM.

The core issues in the field of epilepsy and T1DM were explored using thematic term analysis (Figure 4). Motor themes, located at the top right of the figure, represent the core areas of research. Terms such as “quality of life”, “outcomes”, and “health” reflect the current focus of research. Niche topics, located at the top left, represent highly specialized and peripheral areas of research, such as “acute lymphoblastic leukemia”, “childhood cancer”, and “gender differences”. Basic themes, located in the lower right, include “epilepsy”, “children”, “prevalence”, and “autoantibodies”, which serve as the foundation for building the research framework.

We used Multiple Correspondence Analysis (MCA) to classify the most frequently occurring terms and generated a conceptual structure map (Figure S2), which corresponds to Figure 3. Current research remains focused on the epidemiology of the disease (blue cluster), immune-related mechanisms and comorbidities (red cluster), and clinical outcomes and interventions (blue and green clusters).

Additionally, we performed cluster analysis on the keywords in the literature from 1983 to 2024 using CiteSpace, forming 15 clusters (Figure S3). We visualized the temporal trends of topic occurrences (Figure 4b). In recent years, new trends in the field of epilepsy and T1DM have included “temporal lobe epilepsy”, “status epilepticus”, “risk factors”, “quality of life”, and “management”.

### 3.10. Genetic Overlap Between Epilepsy and T1DM

**Figure 5:**
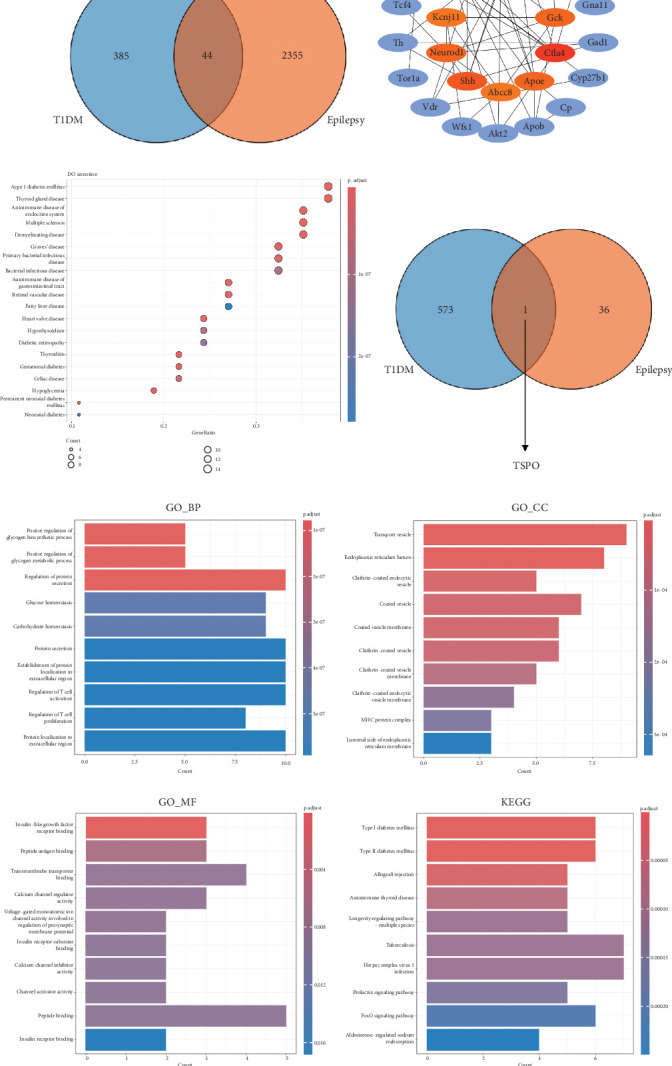
Genetic overlap analysis. (a) Identification of 44 shared genetic targets between epilepsy and T1DM. (b) Visualization of the shared gene network. (c) Enrichment analysis of DO with shared targets. (d) Identification of DEGs between epilepsy and T1DM. (e) GO and KEGG enrichment analyses for the shared genes.

Our genetic analysis identified 429 genes associated with T1DM and 2399 genes associated with epilepsy, with 44 genes shared between the two conditions (Figure 5). This suggests a genuine biological connection rather than an accidental one. Subsequently, the MCC algorithm was used for hub gene analysis, identifying IL10, IGF1, CTLA4, IFIH1, and SHH as the Top 5 genes in the overlapping network (Figure 5 and Figure S4). DO analysis showed that both diseases are closely linked to other conditions, such as autoimmune disease of the endocrine system, thyroid gland disease, and demyelinating disease (Figure 5). KEGG and GO analyses of core genes further explored the major pathways and biological functions, including T1DM, Type 2 diabetes mellitus, allograft rejection, and biological processes such as the regulation of protein secretion, glucose homeostasis, and T cell activation (Figure 5).

Most notably, our analysis of differential gene expression in T1DM (GSE123658) and epilepsy (GSE143272) identified translocator protein (TSPO) as the only gene differentially expressed in both conditions (Figure 5 and Figure S5). TSPO showed upregulation in epilepsy tissue (log2FC = 0.562, *p* < 0.001) and T1DM samples (log2FC = 0.504, *p* = 0.0319). This provides a comprehensive analysis of the genetic link between T1DM and epilepsy, as well as insights into the joint management of these two diseases.

## 4. Discussion

For the first time in this study, we employed bibliometric analysis to conduct an in-depth investigation of 217 publications in the fields of epilepsy and T1DM. We examined various aspects, including countries, institutions, authors, journals, and keywords, and explored the knowledge structure, research hotspots, and emerging trends in this field, presenting our findings visually. Additionally, by integrating data from GeneCards, OMIM, and GEO databases, we explored the genetic intersections between these two conditions and identified 44 shared genes. Notably, TSPO was the only differentially expressed gene. These findings provide a solid theoretical basis for the association between epilepsy and T1DM and offer potential biomarkers and intervention targets for their prevention and treatment.

Over the past decade, academic interest in epilepsy–T1DM comorbidity has grown substantially, particularly regarding shared immune, metabolic, and genetic mechanisms [9]. These factors may be key drivers of the rapid growth of research in this field. Additionally, the rapid development and cost reduction of genome sequencing technology have propelled the advancement of precision medicine and personalized therapy [20, 21]. Building on these advancements and the increased awareness of comorbidity, researchers have begun to focus on the common pathological mechanisms and effective intervention methods underlying epilepsy and T1DM. This focus may be another important reason for the rapid increase in research output in this field. The predicted continued growth trajectory suggests that this field remains in an active development phase, with substantial opportunities for further knowledge advancement.

This rapid growth in research output was not uniform globally. Our analysis of country contributions revealed the United States, the United Kingdom, and China as predominant contributors. These nations demonstrate diverse patterns of international collaboration. The low international collaboration rates observed in some countries (e.g., Norway and China) suggest opportunities for enhanced global cooperation. Given the complex nature of epilepsy–T1DM comorbidity [10–12], international collaborative networks could accelerate knowledge advancement through complementary expertise and larger patient cohorts. In terms of institutional and author contributions, Harvard University has the largest number of publications. Authors Graus F. and Saiz A, from the University of Barcelona, have produced some of the most influential publications in the field, suggesting potential benefits from expanded collaboration with this established research hub. Additionally, high-impact journals such as the *British Medical Journal* and *Brain* have published articles in this field, underscoring its research significance.

In addition to identifying key contributors, our analysis also shed light on the evolving disciplinary landscape of this research area. A dual-map overlay analysis revealed a strong pattern of knowledge flow between disciplines, indicating that research in “molecular/biology/immunology” heavily cites work from “molecular/biology/genetics,” a finding that highlights the foundational role of genetics in the field. Similarly, studies in “medicine/medical/clinical” draw extensively from “health/nursing/medicine” and “molecular/biology/genetics.” This suggests that clinical issues, such as drugs and therapies, are increasingly seeking mechanistic explanations from genetics and are closely linked with broader health management strategies like prevention and nursing. These comprehensive interdisciplinary connections highlight the overall direction, trends, and multidisciplinary integration of the field. Given the complexity of epilepsy–T1DM comorbidity [10–12], interdisciplinary collaboration among neurologists, endocrinologists, immunologists, and geneticists should be strengthened to drive further breakthroughs in understanding this comorbidity.

Although the above analysis provides a macroscopic view, a closer look at the core research themes offers deeper insights. Our analysis suggests that GAD plays a bridging role in both epilepsy and T1DM. GAD, which catalyzes the conversion of glutamate to gamma-aminobutyric acid (GABA), is expressed in both pancreatic *β*-cells and GABAergic neurons, providing a biological basis for the connection [22]. There are two subtypes of GAD: GAD65 and GAD67. Anti-GAD65 antibodies, which are present in approximately 80% of newly diagnosed T1DM patients, have also been detected in various neurological disorders, including stiff-person syndrome, cerebellar ataxia, limbic encephalitis, and certain forms of epilepsy [23–25]. Peltola et al. first documented elevated GAD65 antibodies in patients with therapy-resistant epilepsy [19], while Liimatainen et al. subsequently confirmed this association in a larger cohort [24]. The persistent prominence of GAD-related research in our keyword and co-citation analyses underscores the central role of this autoimmune mechanism in connecting these two conditions [8, 9].

Keyword burst analysis revealed the temporal evolution of research hotspots. Early research focused on antibodies, stiff-person syndrome, and cerebellar ataxia, reflecting initial explorations of autoimmune connections. Subsequent research expanded to epidemiological associations, clinical manifestations, and quality of life considerations. Recent trends indicate growing attention to clinical risk assessment and targeted therapeutic approaches, particularly focusing on the impact of severe hypoglycemia on this comorbidity. Severe hypoglycemia is a common complication among T1DM patients receiving intensive insulin therapy [26]. The International Hypoglycemia Study Group notes that severe hypoglycemia can lead to an increased risk of seizures [11]. Chou et al. showed that the risk of epilepsy in T1DM patients with hypoglycemia was 16.5 times that of the general population and six times that of T1DM patients without hypoglycemia [3]. Maheandiran et al. demonstrated that hypoglycemia can enhance neuronal excitability by affecting potassium channels and the metabolism of excitatory amino acids, thereby inducing seizures [27]. Therefore, for patients with epilepsy and T1DM, continuous glucose monitoring and insulin pump treatment are recommended to reduce the risk of severe hypoglycemia [28, 29]. This is consistent with the clustering results of co-citation analysis.

Building on these bibliometric findings, recent research has increasingly concentrated on the clinical intervention and integrated management of epilepsy and T1DM. In patients with T1DM, both hyperglycemia and hypoglycemia can exacerbate the occurrence of epilepsy [4]. Additionally, recurrent seizures and the use of antiseizure medications can also worsen diabetes [10, 30, 31]. The comorbidity of these two conditions complicates diagnosis and treatment, as well as medication and disease management. The treatment of epilepsy in patients with diabetes primarily involves controlling blood glucose levels, with cautious use of antiseizure medications [32]. For achieving glucose control, insulin is a cornerstone of pharmacological treatment [10]. If the patient continues to experience seizures, antiseizure medications with the minimal impact on blood glucose should be prioritized. In contrast, antiseizure medications such as phenytoin sodium and sodium valproate, which can impair glucose homeostasis, should be avoided [12, 30]. Additionally, in some cases, possible treatment options include immunosuppressive drugs (for GAD65 antibody-positive patients) and a ketogenic diet [8]. For example, one reported case described a significant reduction in seizure frequency in a T1DM patient treated with azathioprine [33]. Similarly, a 10-year study demonstrated that long-term adherence to a low-carb ketogenic diet significantly improved blood glucose control in a patient with T1DM [34]. It should be noted that the ketogenic diet has significant efficacy in patients with refractory epilepsy, but its use in patients with T1DM remains controversial [35], as patients may face high risks for dyslipidemia and hypoglycemia, necessitating close monitoring [36]. Finally, stem-cell therapy represents a promising avenue that may offer new hope to individuals living with both epilepsy and T1DM [37].

While the bibliometric analysis provided a map of the research landscape and highlighted key clinical themes, it can only point toward potential mechanisms indirectly. To investigate the biological connection more directly, we conducted a comprehensive genetic analysis. Our genetic overlap analysis identified 44 shared genes between epilepsy and T1DM, with significant enrichment in immune regulation pathways. The identification of IL10, IGF1, and CTLA4 as top hub genes underscores the importance of immune dysregulation in both conditions. Specifically, IL10 have been associated with both T1DM susceptibility and epilepsy outcomes [38, 39], while CTLA4 variations have been implicated in various autoimmune diseases, including T1DM [40, 41]. These genetic connections provide molecular evidence supporting the autoimmune hypothesis linking epilepsy and T1DM.

Most notably, our analysis identified TSPO as the only gene differentially expressed in both epilepsy and T1DM. TSPO, a transmembrane protein located on the mitochondrial outer membrane, is primarily involved in regulating cholesterol metabolism, oxidative stress, apoptosis, inflammatory responses, and immune functions [42]. It plays a key role in immune regulation and is considered a potential therapeutic target for various diseases, including Alzheimer's disease, multiple sclerosis, cancer, and cardiovascular diseases [43–46]. In the nervous system, TSPO upregulation is a recognized biomarker for neuroinflammation and microglial activation in epilepsy [47]. Studies have shown that TSPO PET imaging can reveal the temporal course and affected brain regions of neuroinflammation, thereby facilitating the diagnosis and treatment of epilepsy [48]. Moreover, recent research has demonstrated that TSPO ligands exhibit anticonvulsant properties in experimental epilepsy models, suggesting their potential therapeutic value in epilepsy treatment [49, 50]. By contrast, research on TSPO in T1DM is relatively limited. However, a recent study using TSPO PET imaging to longitudinally monitor cardiac function in T1DM rat models found that cardiac tissue uptake of the TSPO tracer (18F-FEPPA) significantly increased after the onset of T1DM, consistent with elevated TSPO levels observed in T1DM animal tissues [51]. This finding reveals a novel molecular link between epilepsy and T1DM, indicating that TSPO may serve as a biomarker and potential therapeutic target, warranting further investigation.

## 5. Limitations and Future Perspectives

However, several limitations should be considered when interpreting our findings. First, this study focused on literature published over the past four decades. Consequently, research published in 2025 and beyond will need to be further analyzed by researchers interested in the field. Additionally, our analysis was restricted to the WoSCC, which may contribute to an incomplete literature search. However, bibliometrics is primarily used to analyze trends in a field and to help identify hot topics, so focusing on high-quality publications is appropriate. The WoSCC has strict collection standards, which ensure the overall quality of the collected documents, and is a commonly used reference database in bibliometrics research. Finally, further verification of the findings is lacking. Future studies should explore TSPO expression patterns in comorbid patients, evaluate the therapeutic potential of TSPO ligands for both conditions, and investigate how TSPO-mediated pathways interact with established mechanisms such as GAD autoimmunity.

## 6. Conclusions

We mapped a fast-growing epilepsy–T1DM literature (1983–2024) and uncovered 44 shared genes, with TSPO emerging as a novel, differentially expressed hub. Autoimmunity appears to be the principal mechanistic link. These findings support a common genetic basis and identify TSPO as a potential diagnostic biomarker and therapeutic target. Future work should integrate neurology, endocrinology, immunology, and genetics to improve risk prediction and personalized management of this comorbidity.

## Figures and Tables

**Table 1:** The Top 10 productive countries concerning epilepsy and T1DM research.

**Rank**	**Country**	**Articles**	**Articles (%)**	**SCP**	**MCP**	**MCP (%)**
1	United States	37	17.1	28	9	24.3
2	United Kingdom	23	10.6	15	8	34.8
3	China	15	6.9	14	1	6.7
4	Italy	14	6.5	13	1	7.1
5	France	12	5.5	9	3	25
6	Australia	10	4.6	5	5	50
7	Germany	10	4.6	7	3	30
8	Canada	7	3.2	4	3	42.9
9	The Netherlands	7	3.2	3	4	57.1
10	Norway	7	3.2	7	0	0

Abbreviations: MCP, multiple country publications; SCP, single country publications.

**Table 2:** The most productive 10 authors.

**Rank**	**Author**	**Documents**	**Citations**	**H_index**	**G_index**
1	Graus F.	5	935	5	5
2	Saiz A.	5	1045	5	5
3	Sander J. W.	5	84	4	5
4	Giometto B.	3	153	3	3
5	Liimatainen S.	3	152	3	3
6	Peltola J.	3	366	3	3
7	Rachas A.	3	4	1	1
8	Tuppin P.	3	4	1	1
9	Vianello M.	3	153	3	3
10	Bartolini E.	2	25	2	2

*Note:* Citations are total global citation score.

**Table 3:** The Top 10 papers with the highest number of citations.

**Rank**	**Title**	**Key points**	**Journal**	**Local citations**	**Year**
1	Autoantibodies to Glutamic Acid Decarboxylase in Patients With Therapy-Resistant Epilepsy	Explores the association between GAD autoimmunity and epilepsy, particularly therapy-resistant temporal lobe epilepsy. Highlights the prevalence of GAD antibodies in certain epilepsy cases and their connection to autoimmune processes, aiding in diagnosis and therapeutic approaches.	*Neurology*	27	2000
2	An Association Between Type 1 Diabetes and Idiopathic Generalized Epilepsy	Investigates a potential link between T1DM and idiopathic generalized epilepsy (IGE). Found a four-fold increased prevalence of T1DM in individuals with IGE, suggesting a shared pathophysiological mechanism and a broader disease association with T1DM.	*Annals of Neurology*	26	2006
3	Risk of Epilepsy in Type 1 Diabetes Mellitus: A Population-Based Cohort Study	Analyzes Taiwanese health data to reveal a significantly increased epilepsy risk in patients with Type 1 diabetes. Attributes this to possible metabolic complications like hyperglycemia or hypoglycemia impacting the central nervous system.	*Diabetologia*	25	2016
4	Spectrum of Neurological Syndromes Associated With Glutamic Acid Decarboxylase Antibodies: Diagnostic Clues for This Association	Reviews the diverse neurological syndromes linked to high GAD antibodies, including stiff-person syndrome and cerebellar ataxia. Establishes key demographic and clinical characteristics, highlighting diagnostic relevance for autoimmune neurological syndromes and potential paraneoplastic associations, especially with underlying cancer.	*Brain*	23	2008
5	Type 1 Diabetes Mellitus and Risk of Incident Epilepsy: A Population-Based, Open-Cohort Study	Analyzes British health records to find that individuals with Type 1 diabetes are approximately three times more likely to develop epilepsy compared to nondiabetic counterparts.	*Diabetologia*	21	2017
6	Does Epilepsy Occur More Frequently in Children With Type 1 Diabetes?	Examines the prevalence and types of epilepsy among children with Type 1 diabetes in a large pediatric cohort. Results suggest no higher incidence of epilepsy compared to the general population, though hypoglycemic seizures and EEG abnormalities are common.	*Journal of Paediatrics and Child Health*	20	2008
7	Association of Epilepsy and Type 1 Diabetes Mellitus in Children and Adolescents: Is There an Increased Risk for Diabetic Ketoacidosis?	This study examines the relationship between epilepsy and T1DM in children and adolescents, particularly focusing on whether the occurrence of epilepsy increases the risk of diabetic ketoacidosis (DKA).	*The Journal of Pediatrics*	17	2012
8	Autoantibodies to Glutamic Acid Decarboxylase in Three Patients With Cerebellar Ataxia, Late-Onset Insulin-Dependent Diabetes Mellitus, and Polyendocrine Autoimmunity	Highlights the presence of GAD autoantibodies in patients with cerebellar ataxia and late-onset insulin-dependent diabetes. Demonstrates a strong link between autoimmunity and cerebellar dysfunction, suggesting GAD antibodies as a diagnostic marker for autoimmune-related cerebellar ataxia.	*Neurology*	16	1997
9	Seizures and Type 1 Diabetes Mellitus: Current State of Knowledge	Reviews the potential connection between epilepsy and T1DM. Explores the role of anti-GAD antibodies, which have been implicated in both T1DM and various neurological disorders, including epilepsy.	*European Journal of Endocrinology*	15	2012
10	Population-Level Evidence for an Autoimmune Etiology of Epilepsy	Explores the connection between epilepsy and several common autoimmune diseases using US insurance claim data. Finds robust evidence suggesting an autoimmune component in epilepsy.	*JAMA Neurology*	15	2014

## Data Availability

The data supporting the conclusions of the paper can be found in the paper and supporting information. Other data can be obtained from the corresponding authors upon reasonable request.

## Supporting Information

Supporting Information Additional supporting information can be found online in the Supporting Information section. Figure S1: The PRISMA flow diagram of this study. Table S1: Search strategy. Figure S2: Conceptual structure map. Figure S3: Keyword clusters. Figure S4: MCC algorithm for shared genes centrality analysis. Figure S5: Volcano plot.
