# Supplementary material for: Advancing Understanding of Epilepsy and Type 1 Diabetes Mellitus: A Global Perspective on Research Trends and Future Directions
Source: J Diabetes Res. 2025 Oct 7;2025:8836992. doi: 10.1155/jdr/8836992 (PMC12520817; doi:10.1155/jdr/8836992)

**Fig. S1**

The PRISMA flow diagram of this study


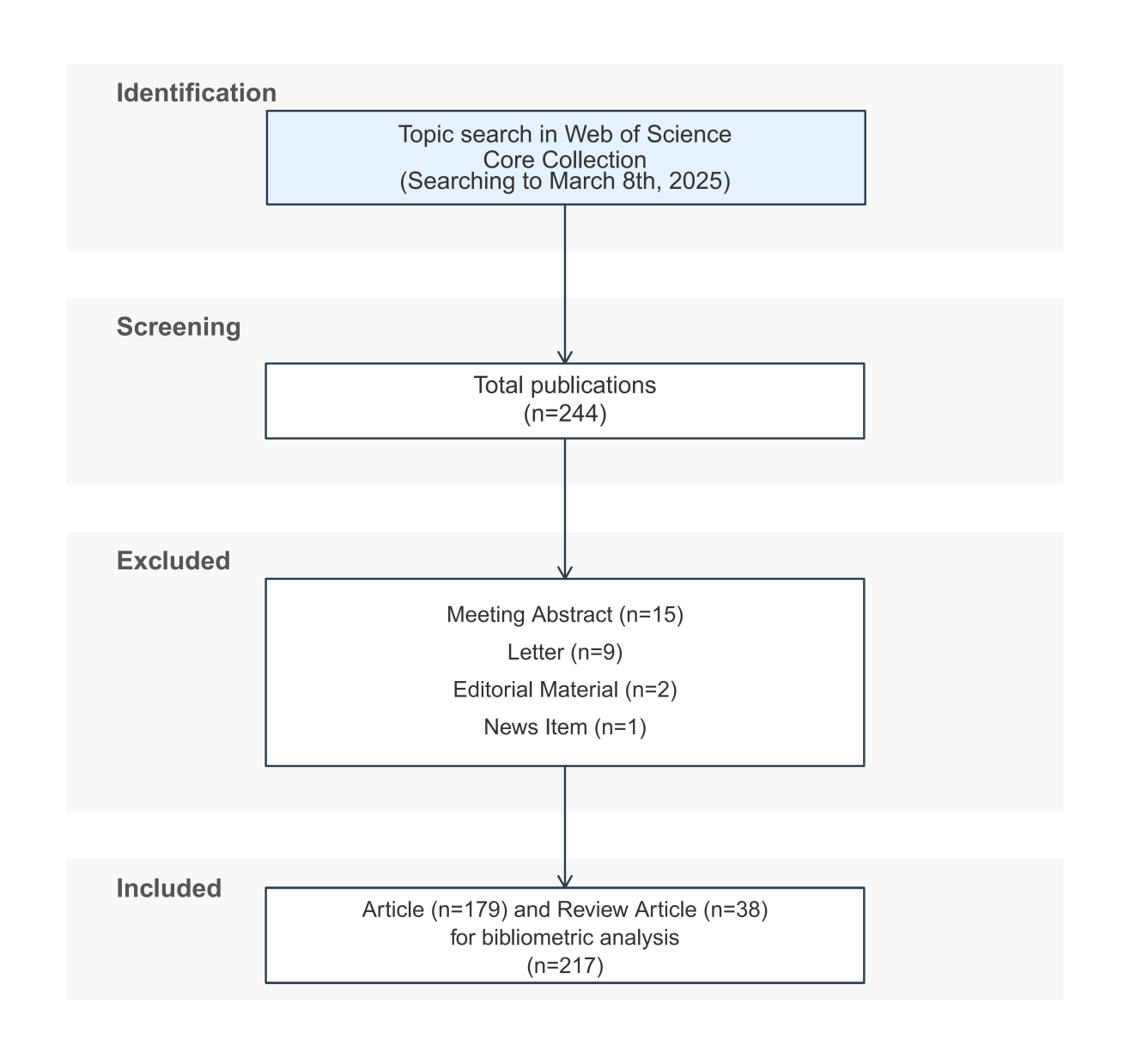


**Table S1**

Search strategy

| Concept | Search Terms |
| --- | --- |
| Concept 1: Epilepsy | Epilepsy OR "Epilepsies" OR "Seizure Disorder" OR "Seizure Disorders" OR "Epilepsy, Cryptogenic" OR "Cryptogenic Epilepsies" OR "Cryptogenic Epilepsy" OR "Epilepsies, Cryptogenic" OR "Aura" OR "Auras" OR "Awakening Epilepsy" OR "Epilepsy, Awakening" |
| Concept 2: Type 1 Diabetes | Diabetes Mellitus, Type 1 OR "Type 1 Diabetes" OR "Diabetes, Type 1" OR "Diabetes Mellitus, Insulin-Dependent" OR "Diabetes Mellitus, Insulin Dependent" OR "Insulin-Dependent Diabetes Mellitus" OR "Diabetes Mellitus, Juvenile-Onset" OR "Diabetes Mellitus, Juvenile Onset" OR "Juvenile-Onset Diabetes Mellitus" OR "IDDM" OR "Diabetes Mellitus, Type I" OR "Diabetes Mellitus, Sudden-Onset" OR "Diabetes Mellitus, Sudden Onset" OR "Sudden-Onset Diabetes Mellitus" OR "Insulin-Dependent Diabetes Mellitus 1" OR "Insulin Dependent Diabetes Mellitus 1" OR "Juvenile-Onset Diabetes" OR "Diabetes, Juvenile-Onset" OR "Juvenile Onset Diabetes" OR "Diabetes, Autoimmune" OR "Autoimmune Diabetes" |
| Combination Method | Concept 1 AND Concept 2 |

**Fig. S2**

Conceptual structure map

**
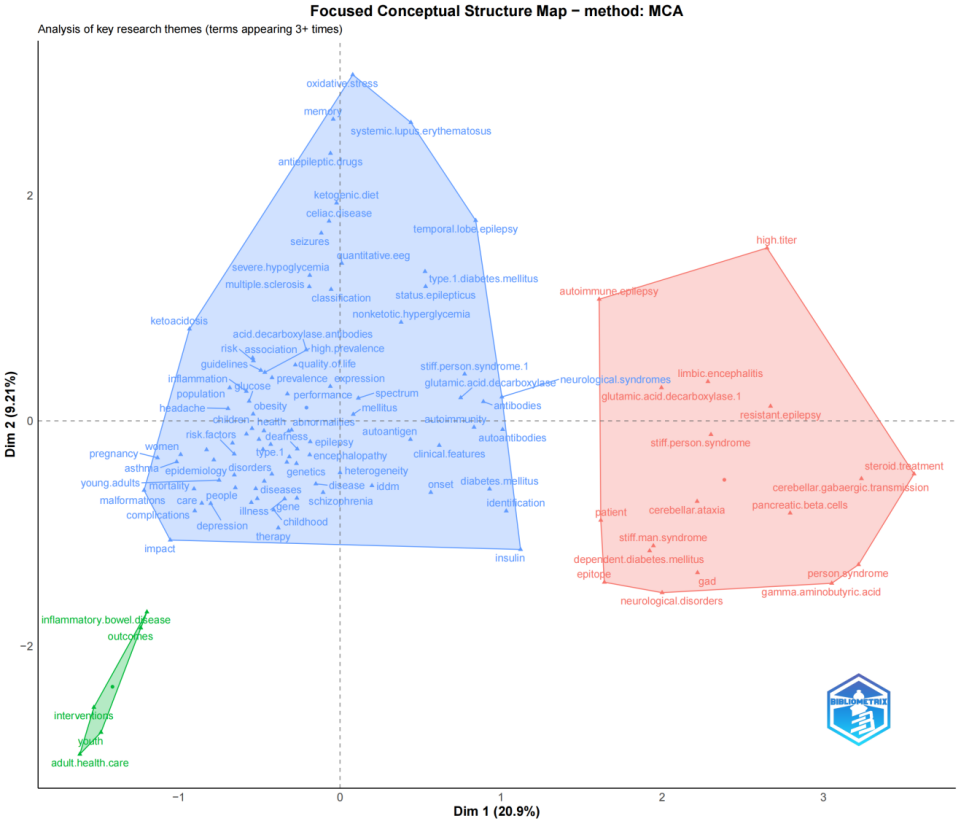
**

**Fig. S3**

Keyword clusters


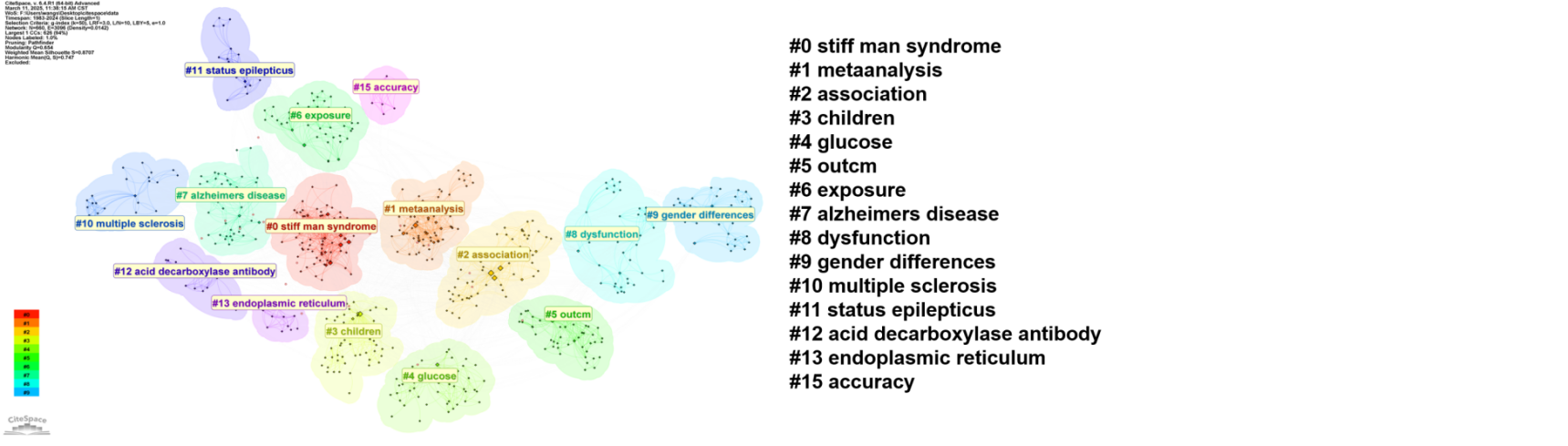


**Fig. S4**

MCC algorithm for shared genes centrality analysis
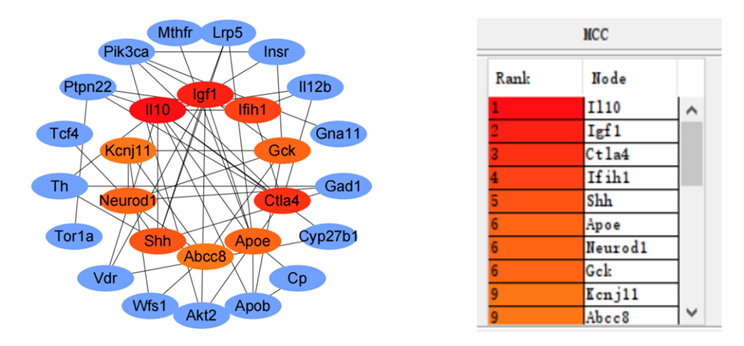


**Fig. S5**

Volcano plot


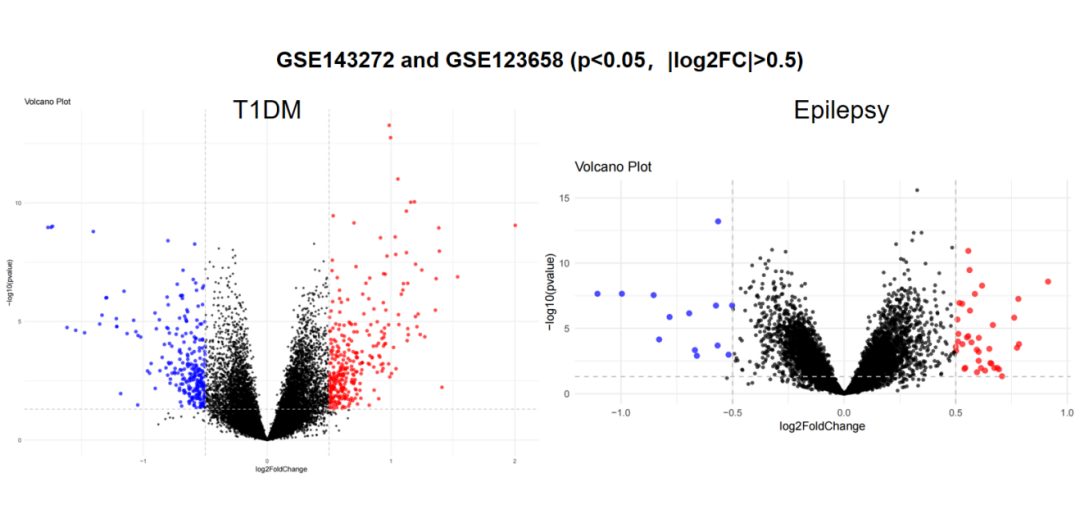

Supplement: Supporting Information — Additional supporting information can be found online in the Supporting Information section. Figure S1: The PRISMA flow diagram of this study. Table S1: Search strategy. Figure S2: Conceptual structure map. Figure S3: Keyword clusters. Figure S4: MCC algorithm for shared genes centrality analysis. Figure S5: Volcano plot. [file 8836992.f1.docx]
